# A Robot Hybrid Hierarchical Network for Sensing Environmental Variables of a Smart Grid

**DOI:** 10.3390/s20195521

**Published:** 2020-09-26

**Authors:** Jiayang Liu, Gongping Wu, Fei Fan, Yuxin Li

**Affiliations:** 1 School of Power and Mechanical Engineering, Wuhan University, Wuhan 430072, China; ljyang@whu.edu.cn (J.L.); gpwu@whu.edu.cn (G.W.); yuxinli@whu.edu.cn (Y.L.); 2 School of Mechanical Engineering and Automation, Wuhan Textile University, Wuhan 430073, China

**Keywords:** hybrid hierarchical network, smart grid, inspection robot, real-time network, optimized deployment, transmission line monitoring

## Abstract

With the rapid development of the social economy, high-voltage transmission lines as power supply infrastructure are increasing, subsequently presenting a new challenge to the effective monitoring of transmission lines. The dynamic sensor network integrated with robots can effectively solve the elastic monitoring of transmission lines, but the problems of real-time performance, energy consumption and economy of the network need to be solved. To solve this problem, a dynamic network deployment method based on the hybrid hierarchical network (HHN) is proposed to realize a low-cost, energy-saving and real-time dynamic sensing system for overhead high-voltage transmission lines. Through case analysis and simulation, combined with the vague set multi-attribute decision-making method (MADM) with scheme preference, the network deployment and optimization results under multi-parameter constraints are obtained.

## 1. Introduction

**Figure 1:**
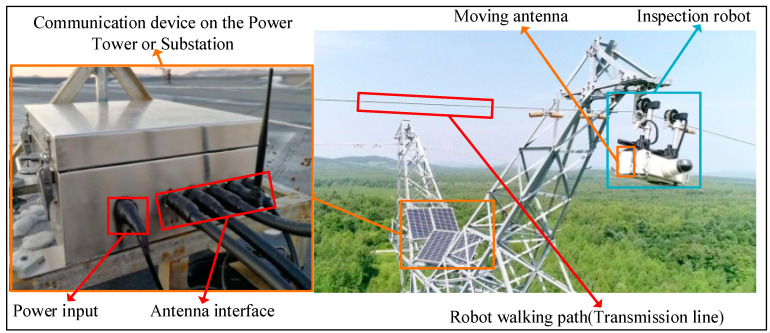
Multi-robot cyber physical system.

With the rapid development of social economy, high-voltage transmission lines as power supply infrastructure are promptly increasing in number. As the transmission lines are usually set up in the field and exposed to a harsh environment for a long time, their operation status monitoring is particularly important. In our previous research [1,2,3,4], a multi-robot cyber physical system (MRCPS), as shown in Figure 1, was proposed to solve the problem of sensing environmental variables of transmission lines.

This novel system combines the characteristics of an inspection robot (IR), wireless sensor networks (WSNs), and a transmission line environment to realize remote monitoring of smart grids [1]. In this system, we adopt the interrupt/delay tolerance technology to realize data elastic transmission. Moreover, network coverage technology improves the delay characteristics of non-real-time network communication. However, transmission lines have long span sections across water areas, randomly distributed tension towers and other key areas. Non-real-time data will affect the continuous monitoring and remote control of robots. Thus, extending MRCPS to construct a regional full-real-time network has become a new challenge.

Nowadays, WSNs are low cost, self-organizing, and dynamic, but their invulnerability in the complex environment needs to be improved [5]. Although 4G/5G communication can provide enough bandwidth, its operation and deployment costs are higher, and there is no signal coverage in remote mountainous areas [6,7]. Optical fiber composite overhead ground wire (OPGW) of smart grids can provide high-speed and long-distance optical fiber communication, but the network expansion performance is limited [8]. Considering the above factors and existing technologies, it is a feasible scheme to construct a hybrid hierarchical network compatible with optical fiber communication and a wireless network.

In this paper, the real-time and reliability of robot inspection and control information are taken as the optimization objectives. Therefore, we focus on the deployment strategy of a dynamic robot hybrid network for smart grids. The research state related to the deployment strategy of a hybrid layered network and its adaption for transmission lines monitoring is as follows.

For transmission line monitoring, multiple fixed sensors are often used to form hybrid multi-layer sensor networks for sensing the state of transmission lines. The deployed sensors will be used to sense the mechanical, physical, or electrical parameters of smart grids, and transmit the data to the monitoring center through the Internet of Things (IoT) [9,10,11]. WSN provides a cost-effective way to quickly establish the communication infrastructure and transmit data from sensors to substations [12,13]. Initially, a small-scale deployment of wireless sensor networks was used to monitor the tension [14], sag [15], and other parameters of transmission lines. For WSN, some towers are not directly connected to the substation and need to send their data to nearby towers closer to the substation. In such a hop-by-hop transmission manner through linear network topology, the data will eventually arrive at the substation. 

However, the wireless network with low bandwidth or data rate limited the monitoring ability [10]. The hybrid hierarchical network is a feasible solution to this problem [16]. It not only retains the flexibility of wireless network deployment, but also improves network performance through a wired network.

A hybrid hierarchical network is a multi-level network structure that integrates multiple communication technologies which have different functions and computing capabilities [17]. The author in [18] proposed to install cellular nodes on each transmission line tower, but this can be prohibitively expensive if there is a large number of towers. To ensure the economy of the network and the timeliness of data, it is very important to deploy the cellular tower and optical fiber-separated towers reasonably. The deployment of its network usually takes into account node energy consumption, cost, end-to-end delay, and network robustness, etc. [19,20,21,22].

Research on hybrid layered network deployment is based on the wide area network (WAN) and WSN. The authors in [23] have formulated a placement problem to determine the number and locations of cellular-enabled towers, such that the installation and operational costs are minimized while satisfying the end-to-end delay and bandwidth constraints of the data stream. Moreover, a linear integer programming model with minimum cost was proposed. However, this method is not suitable for solving nonlinear problems. On this basis, the authors in [24] proposed to use a genetic algorithm to solve the optimal deployment location of network nodes. However, the authors in [23] and [24] have not considered the fact that cellular network coverage may not be available in unpopulated areas. In this case, other forms of WAN, such as satellite communications with universal coverage, should be considered as an alternative to the cellular networks. In [24], considering the requirements of quality of service (QoS) and network robustness, cellular network and satellite network are used to realize communication with the monitoring center. Standard genetic algorithms are used to determine the number, location, and type of WAN connections to be deployed to minimize costs while meeting QoS and robustness requirements.

Research on the hybrid layered deployment network combines the wire network with WSN. The authors of [25] were the first to propose a two-level model, which is especially used to support the application of overhead transmission line monitoring. However, considering the topology constraints of transmission lines, wireless nodes a low bandwidth and low data rate cannot transmit large amounts of data in multi-hop mode. The authors of [26] proposed a reconfigurable network model and studies it from the perspective of security and delay. The goal is to minimize time delays without considering cost and energy constraints. The authors of [27] proposed a theoretical model for the deployment of an optical fiber splitter with the goal of cost and end-to-end delay optimization, and the model was solved by using particle swarm optimization (PSO). This method does not consider the energy consumption of nodes, and it also lacks adaptability in different scenarios. In [20], a network node with optical fiber access is deployed every several towers, and a sequential control scheme is proposed to achieve the best energy efficiency of the nodes, but no specific deployment scheme is given. The authors of [28] introduced the concept of a network partition and proposed a data aggregation point layout algorithm based on clustering to solve the network deployment problem. In this method, the whole network is divided into several sub-nets, and a data aggregation point is deployed in the best location of each sub-net. The authors of [29] proposed a hybrid detection network model based on OPGW and WSN, which mainly studies the power allocation of wireless sensor data transmission. At the same time, the layered network based on the Internet of Things is also widely used in the monitoring of power grid [30,31,32].

To sum up, a hybrid hierarchical network contains a variety of communication modes, and its node deployment is a multi-objective optimization problem. The common methods to determine the optimal deployment location of nodes include linear integer programming, particle swarm optimization, genetic algorithm, etc. At present, few studies have proposed to achieve the real-time monitoring of transmission lines by a robot inspection system based on a hybrid hierarchical network. Furthermore, the existing research has not fully considered the adaptability of the algorithm to the characteristics of transmission lines, such as the number of towers of different sizes. Therefore, we propose a hybrid hierarchical network and its optimization deployment method for MRCPS. It can not only solve the problem of over the horizon real-time communication of inspection robot in key areas, but also realize low-cost and high-efficiency real-time monitoring of transmission lines.

In this article, a robot hybrid hierarchical network (RHHN) and its deployment method are proposed to monitor the state of transmission lines for cyber physical system applications. In addition, the adaptability of the algorithm to the characteristics of transmission lines is fully considered. The main innovations are as follows:

1. A remote real-time communication method based on a hierarchical network for transmission line inspection is proposed.
2. The optimal deployment model of RHHN is given, and the constraints of smart grids are considered.
3. Combining the improved PSO algorithm with the MADM, a specific communication network layout scheme is given.

The structure of this paper is as follows: Section 2 describes the components of RHHN and its deployment model methods. Section 3 discusses the developed methods and algorithms. Simulation work, numerical results and discussions are presented in Section 4, and we conclude the paper in Section 5.

## 2. RHHN and Deployment Model

This section introduces the design and deployment model of RHHN. Firstly, the characteristics of transmission lines are analyzed to establish RHHN. Secondly, the online motion law of the inspection robot is analyzed, and the motion model of the inspection robot is proposed. Thirdly, combined with the motion model, the calculation method of energy consumption and delay in the data forwarding process of the inspection robot is given. Finally, under the constraints of network connectivity, robot endurance, and bandwidth, a deployment model of RHHN is proposed.

### 2.1. Robot Hybrid Hierarchical Network

**Figure 2:**
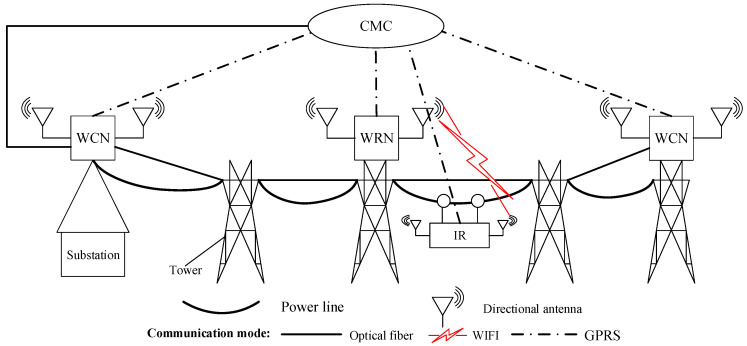
The hybrid hierarchical network communication architecture of the robot hybrid hierarchical network (RHHN).

RHHN integrates OPGW, wireless fidelity (Wi-Fi), and general packet radio service (GPRS) to meet the constraints of data delay, bandwidth, and robot endurance while minimizing deployment costs. Its hybrid hierarchical network communication architecture is shown in Figure 2, which is mainly composed of the following parts:

- Dynamic node: inspection robot (IR).
- Wireless relay node (WRN): communication base station without optical fiber connection equipment.
- Wireless central node (WCN): communication base station with optical fiber connection equipment.
- Central monitoring center (CMC): indoor centralized monitoring platform.

**Figure 3:**
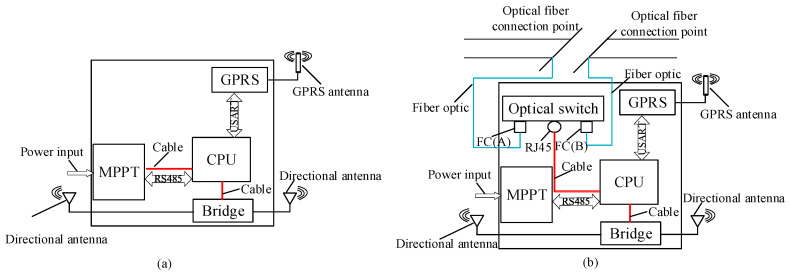
The communication nodes structure: (**a**) wireless relay node structure; (**b**) wireless central node structure.

The communication node (WRNs and WCNs) structures are shown in Figure 3. Their main components include an optical switch, a bridge, GPRS equipment, Maximum Power Point Tracking (MPPT), and a Central Processing Unit (CPU). The CPU controls the whole communication node. MPPT connects solar panels and batteries to realize an automatic power supply. The optical switch provides a stable optical communication link for the network. Then, the network bridge and directional antennas are used to provide directional wireless signals, which cover the transmission line area linearly and provide network access services for other equipment. Moreover, GPRS equipment is configured to obtain the public network access capability in an emergency.

RHHN adopts an optical fiber relay network to form a backbone network. WCN and WRN provide directional wireless signals, which are linear network coverage of transmission line corridors. Therefore, RHHN provides network access services for robots and tower equipment; thus, the inspection data are finally transmitted to the indoor centralized monitoring center. In addition, when the robot is in the wireless coverage blind area and GPRS is available, the robot can use GPRS to feedback to the running state. Furthermore, to prolong the survival period of the node in the harsh environment, RHHN also realizes the sleep scheduling of the communication node through GPRS. The hybrid networking model of integration of OPGW, Wi-Fi, and GPRS expands the access range of OPGW access points and greatly enhances the reliability of the network.

RHHN is divided into three layers, and the specific structure is as follows:

- **Access Layer**

The access layer of the network is responsible for collecting information about the tower. This layer network is composed of the inspection robot (IR) and its inspection payloads. When the robot patrol along the line, the robot can directly transfer the inspection data to the next layer network, or to CMC via GPRS in emergency situation.

- **Distribution Layer**

The distribution layer network is composed of multiple WRNSs, and is responsible for gathering the patrol data sent by the access layer, and then transmitting the patrol data to the core layer in the form of multi-hop. The hop number of data transmission will be determined by wireless link bandwidth and delay tolerance.

- **Core Layer**

The core layer of the network is an optical transport layer. It is composed of CMC and WCN. WCN transmits inspection data to CMC through optical fibers (OPGW). CMC processes and analyzes the inspection data and makes control decisions for the robot.

### 2.2. Related Problem Model

#### 2.2.1. Motion Model of Inspection Robot

**Figure 4:**
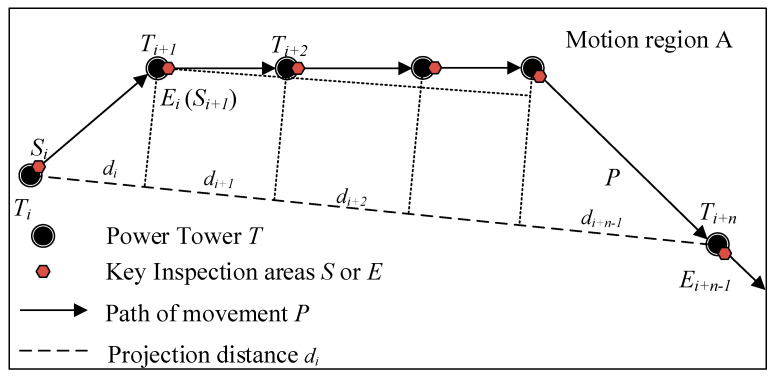
Inspection movement models of robot.

For MRCPS and its RHHN, the inspection robot takes the transmission line as its running track, and the simplified motion law of it is shown in Figure 4. IR has the following three typical motion modes:

- Firstly, line inspection mode: IR moves between towers, that is, it makes a uniform linear motion in each section between towers.
- Secondly, tower crossing motion mode: the robot needs to cross all kinds of hardware near the tower, and makes a low-speed complex movement when it passes through obstacles near the tower.
- Thirdly, power tower inspection mode: after the IR passes over the obstacles, it makes a fine inspection of the area near the tower in a static state.

According to the motion law of the IR, a simplified motion model of the inspection robot and its formula method is given in this section. The motion modeling of the inspection robot is as follows: Suppose that the motion area of the robot is *A*, the path of the robot along the transmission line is *P*, and the transmission line tower is *T*. On any path *P*, the starting point and ending point of the motion are *S* and *E,* respectively. In the interval of *SE*, the robot’s motion speed is *v_IR_* ∈ (*v_min_*, *v_max_*). Among them, the starting point or endpoint of each segment is the key inspection area of the transmission line. That is, the robot carries out the inspection work at *S* (*E*), and during the inspection operation time, the robot keeps still, so as to complete the inspection movement process of the line. After that, the above-mentioned movement process is repeated, and the ending point *E_i_* of the previous movement is taken as the starting point *S*_*i*+1_ of the next movement to form the whole inspection movement.

The robot inspection movement model and the projection distance calculation between the moving endpoints are shown in Figure 4. Suppose that the robot inspects n towers from *T_i_* to *T_i+n_*, the Euclidean distance from *T_i_* to *T_i+n_* is *d_SE_*, and the segmented projection distance is *d_i_*, *d*_*i*+1_, …, *d*_*i* + *n*−1_, (*n*∈N*). When *Rs* = 1, the robot is in the motion state, and the movement rate is *v_IR_* ∈ (*v_min_*, *v_max_*), the duration of robot movement is *t*. When *Rs* = 0, the robot is in the inspection operation state, and the robot remains stationary. Robot inspection mileage *S* (*t*) is shown in Equation (1),

(1) $$S(t)=\left\{ \begin{array}{ll} v_{IR}t & r_{s}=1,v_{IR}\in (v_{\min},v_{\max})\\ 0 & r_{s}=0 \end{array}\right.$$

**Figure 5:**
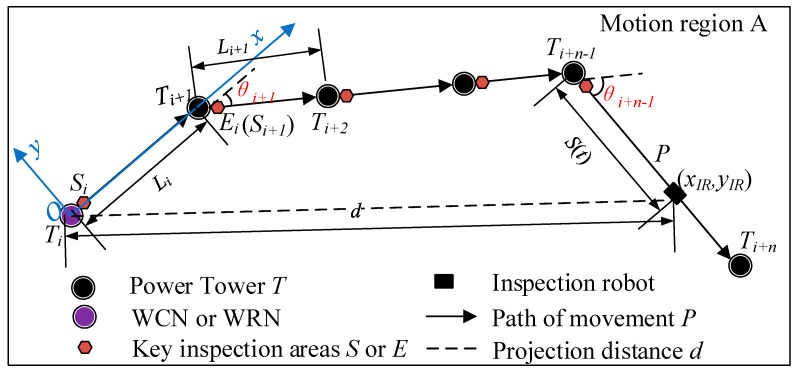
Distance calculation based on robot inspection movement models.

As shown in Figure 5, WCN or WRN is taken as the origin, and the line direction is taken as the *x*-axis coordinate system direction to establish the coordinate system. Then the coordinate of the inspection robot at a certain time is (*x_IR_*, *y_IR_*), which satisfies Equation (2), where L is the distance between the two towers, and S (t) is the distance between the inspection robot and the previous tower, which is calculated by Equation (1). In practical engineering, due to terrain or terrain factors, the direction of the transmission line will change. At this time, the transmission tower at the corner must bear the tension of the transmission line and overhead ground wire. This kind of transmission tower is called an angle-type transmission tower [32]. In Equation (2), θ represents the corner of each tower.

(2) $$\left\{ \begin{array}{l} x_{IR}=L_{i}+\sum_{\varsigma =i+1}^{i+n-2}L_{\varsigma }\cos(\sum_{\kappa =i+1}^{\varsigma }\theta_{\kappa })+s(t)\cos(\sum_{\varsigma =i+1}^{i+n-2}\theta_{\varsigma }+\theta_{i+n-1})\\ y_{IR}=\sum_{\varsigma =i+1}^{i+n-2}L_{\varsigma }\sin(\sum_{\kappa =i+1}^{\varsigma }\theta_{\kappa })+s(t)\sin(\sum_{\varsigma =i+1}^{i+n-2}\theta_{\varsigma }+\theta_{i+n-1}) \end{array}\right.$$

#### 2.2.2. Energy Consumption of Data Transmission 

According to Heinzelman [33], a simplified energy consumption model of data transmission is used for RHHN. The energy consumption of *l* bit data received or transmitted by the node is shown in Equations (3)–(4). Among them, *E_elec_* is the energy consumption coefficient, *d* is the distance between neighboring nodes. The variable $d_{0}\;=\;\sqrt{\varepsilon_{fs}\;/\;\varepsilon_{mp}}$ is the threshold value, the radio free space, and the multi-path loss coefficient are *ε_fs_* and *ε_mp_*, respectively.

(3) $$E_{Rx}=E_{elec}\times l$$

(4) $$E_{Tx}(l,d)=\left\{ \begin{array}{l} lE_{elec}+l\varepsilon_{fs}d^{2},d\leq d_{0}\\ lE_{elec}+l\varepsilon_{mp}d^{4},d>d_{0} \end{array}\right.$$

Equation (4) shows that the farther the neighbor nodes are, the greater the energy consumption in data transmission. Due to the high mobility of the robot, the data transmission energy consumption of RHHN presents dynamic characteristics.

#### 2.2.3. Data Transmission Delay

The communication link delay *D* (*i*, *j*) is a measure of the delay experienced by data from node *i* to node *j*. According to the definition of [10], link delay *D* (*i*, *j*) is shown in Equation (5), including channel access delay *d_ca_*, transmission delay *d_t_* and propagation delay *d_p_*. Where $d_{t}\;=\;l/\lambda$, $d_{p}\;=\;d_{ij}/\Psi$. And *l* (unit: Byte) is the size of the data packet, *λ* (unit: bps) is the link bandwidth, *d_ij_* (unit: m) is the Euclidean distance between node *i* and node *j*, Ψ (unit: m/s) is the propagation speed of the wireless signal in the media, and the average channel access delay *dca = tca*.

(5) $$D(i,j)=d_{ca}+d_{t}+d_{p}$$

The end-to-end delay *D_ete_* (*IR*, *CMC*) of patrol data transmission indicates the time required for data to leave the robot and arrive at CMC. In the process of inspection, the distance between the robot and WRN/WCN changes, so the hop number and delay of data transmission also change. *D_ete_* (*IR*, *CMC*) is a dynamic value, which satisfies Equation (6),

(6) $$\begin{array}{ll} D_{ete}(IR,CMC) & =\sum_{i,j\in \{IR,CMC,W,R\}}D(i,j)\\ & =\sum_{i,j\in \{IR,CMC,W,R\}}\left(t_{ca}+\frac{l}{\lambda }+\frac{d_{ij}}{\psi }\right) \end{array}$$

### 2.3. Deployment Model

To ensure the economic layout and real-time communication, network nodes should be deployed reasonably to optimize the cost and network performance of RHHN. Therefore, we introduce the graph theory to describe RHHN. Furthermore, a node deployment model with path connectivity, robot endurance, and bandwidth constraints is proposed.

#### 2.3.1. RHHN Network Model

**Figure 6:**
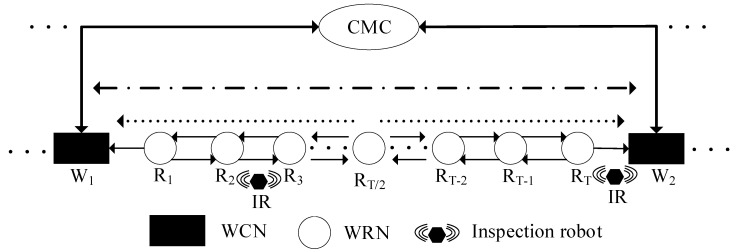
Robot hybrid hierarchical network (RHHN) model.

As shown in Figure 6, the RHHN network model can be represented by a directed graph *G* = (*V*, *E*) [34]. In graph G, the element V represents the vertex set and the corresponding node in RHHN. In actual scenarios, the location of WCN and WRN distribution is unknown, so we need to find a suitable method to solve the deployment strategy of WCN and WRN.

Set V={IR}∪{CMC}∪W∪R, where *W* is the set of WCN; *R* is the set of WRN; and the number of elements in *V* is *N + n,* where the number of WCN/WRN is *N*, and the number of IR/CMC is *n*. The element *E* represents the edge set and represents the physical link between nodes in RHHN. It includes the wired link *l*_Wk, CMC_ = (*W_k_*, CMC) between CMC and WCN; the wireless link *l_IR, Ri_* = (*IR*, *R_i_*) between robot and WRN; the wireless link *l_IR_*_, *Wk*_ = (*IR*, *W_k_*) between robot and WCN; and the wireless link *l_Ri_*_, *Rj*_ *=* (*R_i_*, *R_j_*) between WRN, where *W_k_*, *R_i_* and *R_j_* represent WCN and WRN, respectively, *W_k_*, *R_i_*, *R_j_* ∈ *N*. In the process of robot inspection, the inspection data need to be transmitted to CMC from the robot. These data have common destination and constraints, and each data transmission can be expressed as Equation (7),

(7) F=(Source,Destination,e,b,D)

where *Source* is the source node of the data, that is, IR, and *Destination* is the target node of the data, namely CMC. The variable *e* is the energy consumption of robot the forwarding data, *b* is the bandwidth requirement of data transmission, and *D* is the end-to-end delay of data transmission. 

#### 2.3.2. RHHN Deployment Mode

The RHHN deployment problem is described as follows: under certain constraints, the deployment location of WCN and WRN is determined to balance the network economy and performance.

- **Deployment cost target**

The deployment cost of the whole communication network is mainly the network deployment cost of *N* devices such as WRN and WCN. This is the cost of deploying communication devices on selected transmission line towers. The deployment cost target *f_c_* is shown in Equation (8),

(8) $$f_{c}=\sum_{i=1}^{N_{T}}C_{W}x_{i}+\sum_{j=1}^{N_{T}}C_{R}y_{j}$$

where *N_T_* is the number of transmission towers in a transmission line, and *C_W_* and *C_R_* are the deployment costs of WCN and WRN, respectively. The variables *x_i_* and *y_j_* are deployment coefficients of WCN and WRN, respectively. If power tower *T_i_* deploys WCN, *x_i_* = 1, otherwise *x_i_* = 0. If power tower *T_j_* deploys WRN, then *y_j_* = 1, otherwise *y_j_* = 0.

- **Transmission energy consumption target**

In the process of data transmission, the path selection follows the principle of minimizing energy consumption and delay. In the network model graph *G* = (*V*, *E*), the transmission path *p_IR, CMC_* between the robot and CMC can be described by Equation (9),

(9) $$p_{IR,CMC}=\{l_{IR,i},l_{i,u},\ldots ,l_{w,CMC}\}\subseteq E$$

The energy consumption of robot inspection data forwarding *f_e_* is shown in Equation (10), where neighbor nodes *i*∈ {*W*, *R*}, *E_Rx_* and *E_Tx_* (*l, d_IR, i_*) are the energy consumed by the robot to receive and send information respectively, which are given by Equations (3) and (4). Parameter *l* is the size of the data packet, and parameter *d_IR, i_* is the distance from the robot to the next-hop node. With the movement of the robot, the network changes dynamically, so the energy consumption target of network transmission is changed at different times.

(10) $$f_{e}=E_{Rx}+E_{Tx}(l,d_{IR,i})i\in \left\{W,R\right\}$$

- **Transmission delay target**

Due to the autonomous mobility of the inspection robot, the whole network has local connectivity at different times. Therefore, the network node adjusts the transmission path according to the connectivity characteristics to optimize the real-time performance. Different transmission paths *p_IR, CMC_* lead to the change in hop number (*h* (*p_IR, CMC_*) = |*p_IR, CMC_*| can be used to represent the hop number of data link) and distance *d_IR, CMC_* of patrol data transmission. The maximum end-to-end delay of data transmission in RHHN can be obtained by combining Equations (6) and (9). The transmission delay target is shown in Equation (11), where *d_IR, i_* is the distance from the robot to the next-hop node.

(11) $$f_{d}=[\max(h(p_{IR,CMC}))+1](t_{ca}+\frac{l}{\lambda })+\frac{l}{\lambda }+\frac{d_{IR,i}}{\psi }i\in \left\{W,R\right\}$$

- **Multi-objective function**

Considering the multi-objective matching and optimization problem of RHHN deployment cost, network transmission energy consumption, and network real-time performance, the RHHN multi-objective function is shown in Equation (12),

(12) $$\min F=[f_{c},f_{e},f_{d}]$$

In the communication network deployment for MRCPS, the objective function *f_c_* is discontinuous and *f_e_* and *f_d_* are nonlinear functions. Therefore, the problem of RHHN deployment can be defined as a discontinuous, nonlinear, multi-variable, and multi-objective optimization problem.

- **Constraint condition**

The system data are transmitted to WCN in the form of multi-hop through RHHN and then uploaded to CMC from WCN through optical fibers (OPGW). As the data are transmitted in the network, whether the data of node i pass through a certain link can be described by the variable *X_i, j, k_*, where *i* and *j* in the variable denote the start and end nodes of the link, respectively. If the patrol data use the link, the value of *X_i, j, k_* = 1, otherwise it is 0. In order to ensure the connectivity of data transmission path, according to the geometric relationship in graph *G* = (*V*, *E*), the constraint conditions are established as follows,

(13) $$\sum_{k=1}^{N+1}\sum_{i=1}^{W}X_{i,CMC,k}=N+1$$

(14) $$X_{i,CMC,k}=1\forall i\in W,k\in N+1$$

(15) $$X_{i,j,k}\leq X_{j,CMC,k}\forall i\in R\cup IR,j\in W,k\in [1,N+1]$$

(16) $$X_{i,CMC,k}-x_{i}\leq 0\forall i\in W,k\in [1,N+1]$$

(17) $$x_{i},y_{j},X_{i,j,k}\in \{0,1\}\forall i,j,k$$

Equations (13) and (14) ensure that system data must arrive at CMC through WCN and the destination of the data is CMC, where W is the set of WCN. Then, Equation (15) ensures that the data first reach WCN and then forwards to CMC. Equation (16) ensures that WCN is installed on the required power tower *T_i_* when any data stream uses the link. Finally, Equation (17) ensures that the decision variables are binary variables with values of 0 and 1. 

Furthermore, the constraints of network delay, bandwidth, and robot energy consumption should be considered in network planning. Equation (18) ensures that the delay of system data transmission from IR to CMC is less than or equal to the maximum allowable end-to-end delay *D_ete_* (*IR, CMC*). As the robot patrols, the hop number *h* (*p_IR, CMC_*) of data transmission also changes with the change of robot position. When the robot runs to the midpoint of the two WCNs, the hop number *h* (*p_IR, CMC_*) of data transmission is the maximum, and *f_d_* is the maximum value. Therefore, the maximum value of the transmission delay target *f_d_* can be calculated by calculating the maximum value of *h* (*p_IR, CMC_*). Equation (19) ensures that the total traffic on each link does not exceed the available bandwidth of the link. Where *b_k_* is the data generation rate of node *k*, then *B_i, j_* is the bandwidth constraint of link *L* (*i, j*). 

Equation (20) is the constraint of robot communication energy consumption. It indicates that at time *t*, the total energy of the robot is *E_IR_* (*t*), and the energy consumed by the robot to transmit data through the link *L* (*IR, i*) is *e_IR, i_* (*t*).

(18) $$f_{d}\leq D_{ete}(IR,\;CMC)$$

(19) $$\sum_{k\in N+1}b_{k}X_{i,j,k}\leq B_{ij}\forall (i,j)\in E$$

(20) $$\sum e_{IR,i}(t)<E_{IR}(t)\forall i\in W\cup R$$

**Figure 7:**
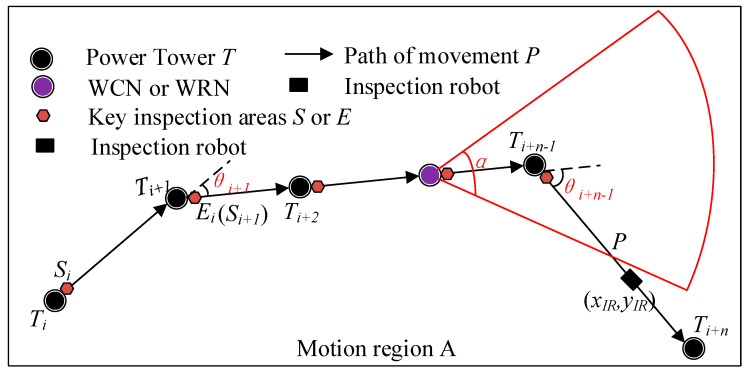
Effective coverage area of the linear wireless signal.

There are many corners in the transmission line. When the corners are too large, the effective coverage area of the linear wireless signal is greatly reduced, as shown in Figure 7. In order to maximize the effective coverage area of linear signals at communication nodes and ensure the effective connection between nodes, the node deployment meets the constraint of Equation (21). Equation (21) indicates that WCN or WRN is deployed at tower *i* when the line angle at tower *i* is greater than or equal to α/2. Where *N_T_* is the number of transmission line towers, *θ* is the corners of each tower, and *α* is the horizontal lobe angle of the antenna.

(21) $$\left\{ \begin{array}{l} x_{i}=1\;\forall i\in N_{T},\theta_{i}\geq \alpha /2\\ y_{j}=1\;\forall j\in N_{T},\theta_{j}\geq \alpha /2 \end{array}\right.$$

## 3. The Deployment Method

In Section 2, we prove that the RHHN deployment problem is a discontinuous, nonlinear, multi-variable, and multi-objective optimization problem. Firstly, we analyze the relationship between network cost, delay, and energy consumption in this Section. Then, the three-objective optimization model is transformed into two simplified two objective optimization models, and the improved PSO is used to solve the deployment scheme. Finally, the MADM of a vague set with preference is used to sort the deployment schemes and determine the optimal deployment scheme to meet the needs of actual scenarios [35].

### 3.1. Transformation Model

In RHHN, the inspection data are first forwarded to the communication node by the mobile robot nearby. After receiving the inspection data, the node forwards the data to WCN through multi-hop transmission. Finally, WCN transmits the inspection data to CMC through OPGW. 

It can be seen that the energy consumption in the process of data forwarding is only related to the distance *d_IR, i_* between the robot and the communication node. Moreover, it is independent of the type of node (WCN or WRN). The end-to-end delay *D_ete_* (*IR, CMC*) of data transmission represents the time required for data to be forwarded from the robot to CMC. The value of *D_ete_* (*IR, CMC*) is positively correlated with the number of hops and the distance *d* between nodes. Then, when the WRN position is known, the value of *D_ete_* (*IR, CMC*) only depends on the number of data forwarding hops *h* (*p_IR, CMC_*). In addition, the value of *h* (*p_IR, CMC_*) depends on the deployment plan of WCN. 

Therefore, the three objective optimization problems can be transformed into two double objective optimization problems. In other words, the deployment planning of WRN is solved by Equation (21). After obtaining the deployment scheme of WRN, the WCN deployment plan is solved by Equation (22).

(22) $$\left\{ \begin{array}{l} f_{c_{R}}=\sum_{i=1}^{N_{T}}C_{R}y_{i}\\ f_{e}=E_{Rx}+E_{Tx}(l,d_{IR,i}) \end{array}\right.$$

(23) $$\left\{ \begin{array}{l} f_{c_{W}}=\sum_{i=1}^{N_{T}}C_{W}x_{i}\\ f_{d}=\sum_{i,j\in \{IR,CMC,W,R\}}\left(t_{ca}+\frac{l}{\lambda }+\frac{d_{ij}}{\psi }\right) \end{array}\right.$$

In the multi-objective optimization problem, optimizing one of the objectives must sacrifice the other as the cost, so there is no single optimal solution for the overall goal. However, in practice, there are multiple optimal compromise solutions, which are called Pareto optimal solutions [36]. Usually, the multi-objective optimization problem can be transformed into a single objective optimization problem by weighting the objective, and then solved by mathematical programming method. However, this weighted serialization method can only obtain one optimal solution at a time. At present, the method to solve multi-objective optimization problem is the multi-objective evolutionary algorithm [37]. In the multi-objective evolutionary algorithm, the PSO algorithm has the characteristics of fast convergence speed and simple operation. In this paper, an improved PSO algorithm is used to solve the Pareto optimal solution set of objective function. In addition, a vague set multi-attribute decision-making method with a preference for alternatives is proposed to rank the optimal solution set. This method determines the optimal solution according to the actual scene, which makes up for the blindness of linear weighting.

### 3.2. Improved PSO for RHHN Deployment

Particle swarm optimization [38] was proposed by Kennedy and Eberhart in 1995. After that, Shi [39] introduced the inertia weight factor on this basis, which is called the basic particle swarm optimization algorithm. The update equation of inertia weight *w* and particle velocity is as follows:

(24) $$\left\{ \begin{array}{l} w=w_{\max}-(w_{\max}-w_{\min})/(MaxIt-1)\cdot iter\\ v_{ij}(t+1)=wv_{ij}(t)+c_{1}r_{1}(t)(p_{ij}(t)-x_{ij}(t))+c_{2}r_{2}(t)(p_{gj}(t)-x_{ij}(t)) \end{array}\right.$$

where *w_max_* and *w_min_* are the maximum and minimum values of inertia weight, respectively. The variables *MaxIt* and *iter* are the maximum number of iterations and the current number of iterations, respectively. Parameters *c*_1_ and *c*_2_ are learning factors. Parameters *r*_1_ and *r*_2_ are random numbers with uniform distribution on [0, 1].

However, the linear inertia weight of PSO has no emphasis on global search and local search. In addition, the PSO algorithm needs to conduct a wide range of global search in the initial stage. In the later stage, it needs strong local searchability, and at the same time, it needs to speed up the convergence speed of the algorithm. In this paper, a nonlinear sine function curve is introduced to adjust the inertia weight *w* of PSO. The slope of the sine function curve increases first and then decreases, which makes particles focus on global search first and then local search. At the same time, adjusting the update mode of learning factors *c*_1_ and *c*_2_ is helpful for particle swarm optimization to quickly search the global optimum. The specific improvement of the improved PSO (SinPSO) algorithm is shown in Equation (25):

(25) $$\left\{ \begin{array}{l} w=\frac{w_{\max}-w_{\min}}{2}\cos\frac{iter\cdot \pi }{MaxIt}+\frac{w_{\max}+w_{\min}}{2}\\ c_{1}=c_{1\max}-\frac{(c_{1\max}-c_{1\min})\cdot iter}{MaxIt}\\ c_{2}=c_{2\min}+\frac{(c_{2\max}-c_{2\min})\cdot iter}{MaxIt}\\ v_{ij}(t+1)=wv_{ij}(t)+c_{1}r_{1}(t)(p_{ij}(t)-x_{ij}(t))+c_{2}r_{2}(t)(p_{gj}(t)-x_{ij}(t)) \end{array}\right.$$

In the deployment of RHHN, it is necessary to optimize the specific installation location and deployment number of WRNs and WCNs. Therefore, the particle is a coding sequence formed by *x_i_*, which represents the installation position of WRNs and WCNs. The encoding form is shown in Equation (26). Different particles constitute a heterogeneous deployment scheme. If it is WCN or WRN, the corresponding element in the coding sequence is 1, otherwise it is 0.

(26) $$X=[x_{1},x_{2},\ldots ,x_{N}]$$

The pseudo-code of SinPSO is shown in Algorithm 1 as follows.
**Algorithm 1**: SinPSO1: **for** each particle *i*2:   Initialize velocity *vi* and position *Xi* for particle *i*3:   Evaluate particle *i* and set *pBesti* = *Xi*4: **end for**5: *gBest* = min{*pBesti*}6: **for** i = 1 to MaxIt7:   Evaluate particle *i*8:   **if** fit (*Xi*) < fit(*pBesti*)9:   *pBesti* = *Xi*;10:  **end if**11:  **if** fit(*pBesti*) < fit(*gBest*)12:    *gBest* = *pBesti*;13:  **end if**14:  for each particle *i*, update *vi* and position *Xi* according to Equation (25)15: **end for**16: print *gBest*

### 3.3. Multi-Attribute Decision Making 

After the Pareto optimal solution set is obtained by SinPSO, the optimal solution set is sorted by the multi-attribute decision-making method (MADM) of a vague set with a preference on alternatives. The optimal solution set is sorted by MADM of a vague set with a preference on alternatives, and its specific steps are as follows:

- **Step 1:** Calculate the vague value.

Using Equation (27), each attribute index is standardized. Then, according to the size of variable *r_ij_*, the corresponding Vague value *a_ij_* = [*g_ij_*, 1 − *m_ij_*] of scheme A*_i_* under the *j-th* evaluation index is obtained. Where g*_ij_* is the true membership of vague sets, m*_ij_* is the false membership of vague sets, and *g_ij_*, *m_ij_* ∈ [0, 1]. The decision maker’s preference for scheme *A_i_* is expressed by vague as a_i_* = [g_i_*, 1 − m_i_*]. The corresponding relationship between the size of variable *r_ij_* and vague value is shown in Table 1.

(27) $$r_{ij}=\frac{x_{j\min}}{x_{ij}},x_{j\min}=\underset{i}{\min}x_{ij}$$

- **Step 2:** Calculate weight.

The weight *x_j_* of each attribute is calculated according to Equation (28), where ξ and ς represent the number of programs and evaluation indicators, respectively.

(28) $$\chi_{j}=\frac{\sum_{i=1}^{\xi }\left(|g_{ij}-g_{i}{}^{*}|+|m_{ij}-m_{i}{}^{*}|\right)}{\sum_{j=1}^{\zeta }\sum_{i=1}^{\xi }\left(|g_{ij}-g_{i}{}^{*}|+|m_{ij}-m_{i}{}^{*}|\right)}$$

- **Step 3:** Calculation of the comprehensive attribute value.

The comprehensive value *Z_i_* = [*g_i_*’, 1 − *m_i_*’] of each scheme attribute is calculated according to Equation (29), where *g_i_*’ and *m_i_*’ are the comprehensive values of true membership and false membership of scheme *A_i_*, respectively.

(29) $$Z_{i}=\sum_{j=1}^{\zeta }a_{ij}\chi_{j}$$

- **Step 4:** Calculation of the possibility matrix.

According to Equation (30), the possibility degree of comparison among the comprehensive values of the attributes of each scheme is calculated, and the possibility matrix *P* is obtained.

(30) $$P(Z_{i}>Z_{j})=\frac{\max[0,(1-m_{i}{}^{\prime }+1-m_{j}{}^{\prime }-g_{i}{}^{\prime }-g_{j}{}^{\prime })-\max(0,1-m_{j}{}^{\prime }-g_{i}{}^{\prime })]}{1-m_{i}{}^{\prime }+1-m_{j}{}^{\prime }-g_{i}{}^{\prime }-g_{j}{}^{\prime }}$$

- **Step 5:** Comprehensive ranking calculation of schemes.

The ranking vector *v* of possibility degree is calculated from the possibility degree matrix P, and the comprehensive ranking of alternatives is obtained. Mostly, *v* [*i*] is compared, and the largest one is the selection scheme. If *v* [*i*] is the same, the scheme with a larger *g_i_*’ value is selected.

In the actual scenario, the number of transmission lines is not an integral multiple of WRN or WCN. In general, a balanced deployment of WRN and WCN can achieve optimal network performance. Therefore, when the cost-robot energy consumption or cost-delay are consistent, the deployment scheme with the minimum variance should be selected. The variance is calculated as Equation (31), where *d_i_* is the distance between two communication nodes, *n* is the number of communication nodes, and *S* is the length of the transmission line.

(31) $$\sigma^{2}=\frac{1}{n}\sum_{i=1}^{n}{\left(d_{i}-\frac{S}{n}\right)}^{2}$$

## 4. Results and Discussion

In the multi-objective optimization problem, the higher the objective dimension, the more serious the pressure attenuation of the excellent solution selection. In addition, it is difficult to maintain the convergence and diversity of the objective solution set at the same time [40]. Generally, dimensionality reduction is an effective method to improve the diversity and convergence of solutions. Meanwhile, this method can reduce the complexity of the algorithm and improve computational efficiency. Therefore, in Section 4, we analyze the relationship between network cost, robot energy consumption, and data transmission delay. Then, the three-objective optimization problem is transformed into two double objective optimization problems.

In this section, we try to use the two-dimensional objective optimization method to implement the actual deployment of RHHN. Furthermore, SinPSO is compared with several deployment algorithms to evaluate its efficiency. The energy consumption in the process of data forwarding is only related to the distance between the robot and the communication node. It has nothing to do with the type of node (WCN or WRN), while the network delay *D_ete_* (*IR, CMC*) is positively correlated with the number of relay hops *h* (*p_IR, CMC_*) and the distance *d* between nodes. When the deployment scheme of WRN is not determined, the value of *h* (*p_IR, CMC_*) cannot be determined. Therefore, it is very important to obtain the deployment scheme of WRN by solving the two-dimensional optimization problem of deployment cost and robot energy consumption. To sum up, the deployment of RHHN communication node is divided into the following steps:

- Step 1: The optimal objective function of WRN deployment cost and the robot energy consumption is solved to obtain the deployment scheme of WRN.
- Step 2: According to the relationship between the maximum end-to-end delay of data transmission and the deployment cost of WCN, the deployment scheme of WCN is solved.

Thus, combined with the WRN deployment scheme and the WCN deployment scheme, the final deployment strategy is obtained.

### 4.1. Deployment of WRNs 

In order to verify the effectiveness of the proposed RHHN deployment model and algorithm, SinPSO is used to solve the deployment problem. In the scenario of different numbers and scales of towers, the solution set of SinPSO algorithm is compared with that of the non-dominated sorting genetic algorithm-II (NSGA-II) [41] and standard PSO [39]. The reason why the NSGA-II algorithm is selected for comparison is that the NSGA-II algorithm is widely recognized as a classic algorithm for multi-objective optimization. The comparison with PSO algorithm is to reflect the advantages of SinPSO algorithm.

For simulation scenarios, the size of data package received by the robot is 30 bit and the size of data package sent is 1200 Kb during each data transmission process. All nodes have wireless communication capabilities similar to IEEE 802.11n, and the transmission rate is 10 Mbps. The default network transmission range of WCNs, WRNs and robot is 8 km. The horizontal lobe angle of directional antenna is 60°. Considering installation, equipment procurement and other factors, the deployment costs of WCN and WRN are 7000 (USD) and 3500 (USD), respectively. The distance between towers is 100 m to 1000 m, but it is a definite number. SinPSO, NSGA-II and PSO were used to solve the deployment of experimental scenarios with the number of towers of 50, 100, 200, 300 and 500, respectively, and 100 simulations were carried out for each scenario. The default simulation parameters are shown in Table 2. The basic parameters and parameter values of each algorithm are shown in Table 3 and Table 4. The learning factor of the basic PSO algorithm is not updated during the iteration process and is set to a fixed value *c*_1_ = *c*_2_ = 0.55.

**Figure 8:**
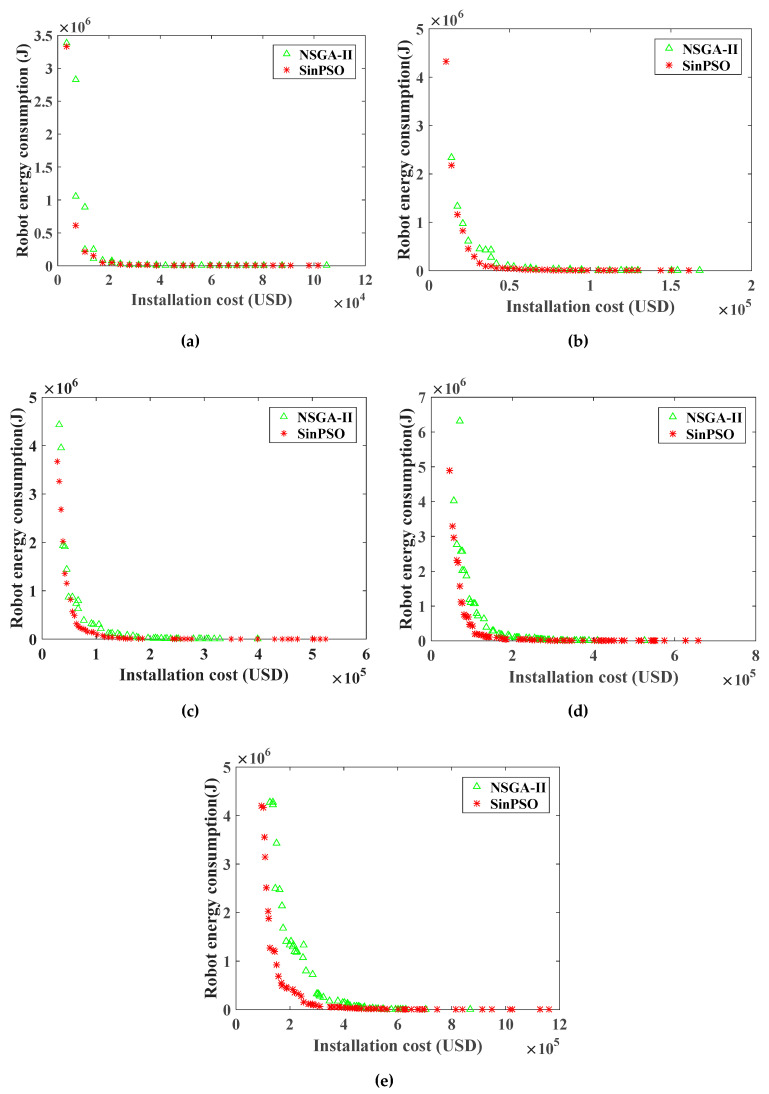
Pareto frontier comparison of deployment cost and optimization objective function of robot energy consumption for WRN by NSGA-II and SinPSO: (**a**) the number of towers is 50; (**b**) the number of towers is 100; (**c**) the number of towers is 200; (**d**) the number of towers is 300; (**e**) the number of towers is 500.

**Figure 9:**
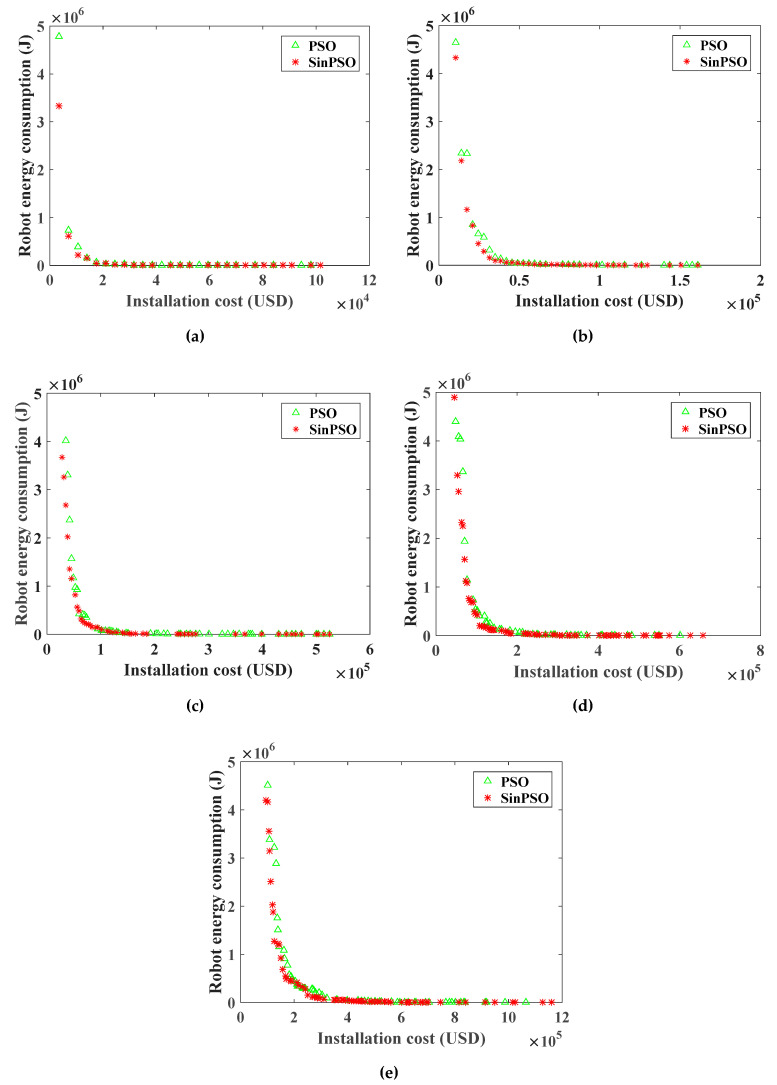
Pareto frontier comparison of deployment cost and optimization objective function of robot energy consumption for WRN by PSO and SinPSO: (**a**) the number of towers is 50; (**b**) the number of towers is 100; (**c**) the number of towers is 200; (**d**) the number of towers is 300; (**e**) the number of towers is 500.

For the RHHN deployment model, the Pareto frontier of the optimization objective function of WRN installation cost and the robot energy consumption is shown in Figure 8 and Figure 9. Figure 7 shows the Pareto front comparison of NSGA-II and SinPSO with tower numbers of 50, 100, 200, 300 and 500, respectively. Besides, Figure 8 shows the Pareto front comparison between SinPSO and standard PSO.

Analyzing the Pareto frontier contrast diagrams of Figure 8 and Figure 9 shows that with the increase in deployment cost, the number of nodes in the network gradually increases. With the increase in the number of nodes, the distance between nodes decreases, and the energy consumption in the process of data forwarding is less. By increasing the deployment cost, the energy consumption in the process of data transmission can be effectively reduced. Besides, the Pareto solution obtained by SinPSO is better than NSGA-II when the number of transmission lines is 50, 100, 200, 300 and 500. With the same deployment cost, the energy consumption of the robot is smaller. Under the same robot energy consumption, the node deployment cost is lower. Similarly, it can be seen from Figure 9 that the PSO algorithm is much better than NSGA-II. However, the solution set of SinPSO with nonlinear inertia weight is better than that of basic PSO. Analyzing the experimental data of Figure 8 and Figure 9, we can see that SinPSO has obvious advantages over NSGA-II and PSO when the deployment rate of communication node Γ ($\Gamma \;=\;\frac{n_{t}}{N_{T}}$, $n_{t}$ is the number of communication nodes, and $N_{T}$ is the number of towers) is less than 0.3. With the increase in Γ, the advantage of SinPSO decreases. This is because the number of deployed communication nodes increases with the increase in Γ, and the distribution of communication nodes is more uniform. In the experiments with 50, 100, 200, 300, and 500 transmission line towers, compared with the deployment scheme solved by NSGA-II, the robot energy consumption in the deployment scheme solved by SinPSO was reduced by 76.1%, 78.5%, 66.3%, 74.3% and 77.8%, respectively, at the same deployment cost. Similarly, compared with the deployment scheme solved by PSO, under the same deployment cost, the robot energy consumption in the deployment scheme solved by SinPSO was reduced by 30.2%, 50.0%, 42.8%, 33.0% and 41.2%, respectively.

**Figure 10:**
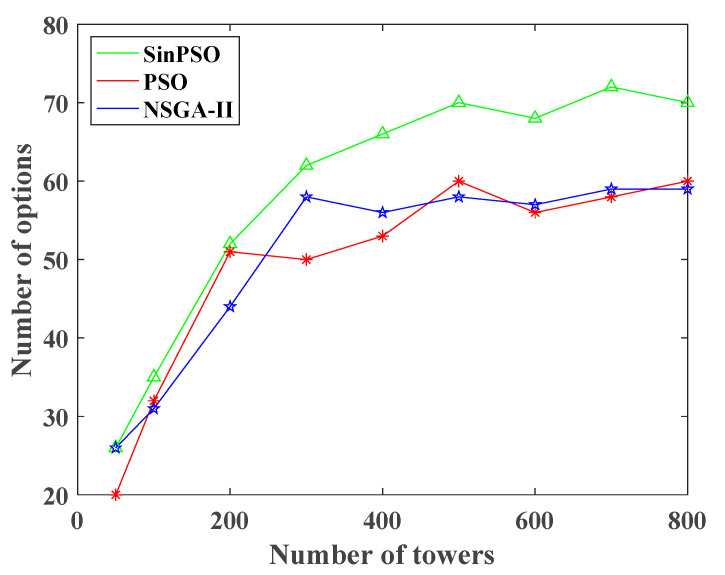
The result of algorithm diversity.

It is found that when the number of towers increases from 300 to 500, the number of solution sets of SinPSO algorithm increases significantly. Therefore, the simulation experiment of 400, 600, 700, and 800 is added. As shown in Figure 10, the diversity of SinPSO solutions is better than that of PSO and NSGA-II under the same sample size, which provides more options for decision makers. When the sample size is more than 500, the diversity of solution sets of the three algorithms tends to be stable. Thus, the solution set obtained by SinPSO has better convergence and diversity.

To measure the computational complexity, the running time of each algorithm is compared and analyzed. The average time of the WRN optimization function running for 100 times under different number of power towers is counted, as shown in Table 5.

On the whole, the running time of NSGA-II is significantly longer than that of SinPSO and PSO. With the increase in the number of towers, the running time of the three algorithms is increasing. In the process of optimization, SinPSO first focuses on global search, then focuses on local search, which is conducive to particle swarm optimization to quickly search the global optimal. Therefore, the running times of SinPSO and PSO are basically the same, and SinPSO is slightly better than PSO. To sum up, SinPSO has lower algorithm complexity than PSO and NSGA-II.

**Figure 11:**
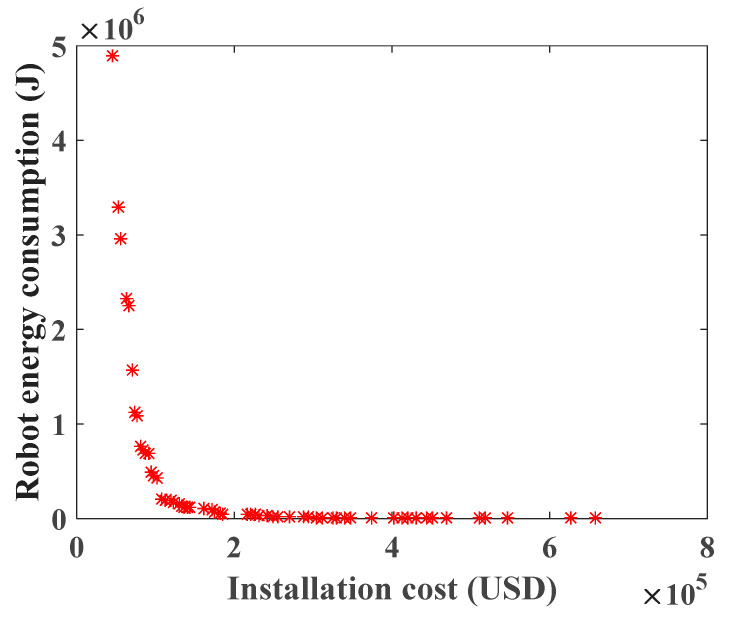
Pareto front with 300 towers.

After the objective function of WRN deployment is obtained, the comprehensive score of each scheme is calculated by the multi-attribute decision-making method in Section 3.3. Taking the scenario with 300 towers as an example, the Pareto front of Equation (22) is obtained by SinPSO, as shown in Figure 11.

**Figure 12:**
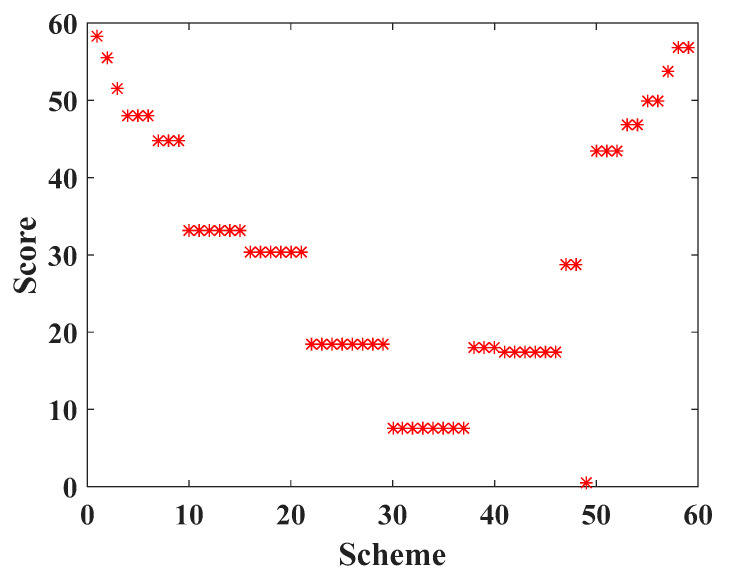
Comprehensive attribute score of each deployment scheme of WRN.

Among the 59 schemes in Figure 11, the score chart of each scheme is shown in Figure 12, and the scheme with the highest score is Scheme 1. For Scheme 1, the deployment cost of WRN is USD 45,500, and the energy consumption of the robot is 4,881,927.16041553 J. According to Equation (30), the uniform layout scheme is selected. The specific deployment of WRNs and its related parameters are shown in Table 6.

### 4.2. Deployment of WCNs 

Taking 300 towers as an example, the optimal solution set of objective function is obtained twice, and the MADM of vague set with preference for alternatives is used to make decision. After obtaining the layout scheme of Equation (22), the optimal solution set of Equation (23) is obtained by using SinPSO, NSGA-II and PSO. As shown in Figure 10, the maximum end-to-end delay is compared with the deployment cost planning scheme. The input parameters are as follows: wireless signal propagation speed: 3.0 × 10^8^ m·s^−1^; average channel access delay: *t_ca_* = 41 ms; WCN cost: *C_w_* = USD 7000; link bandwidth limit: *B_ij_* = 10 Mbps.

**Figure 13:**
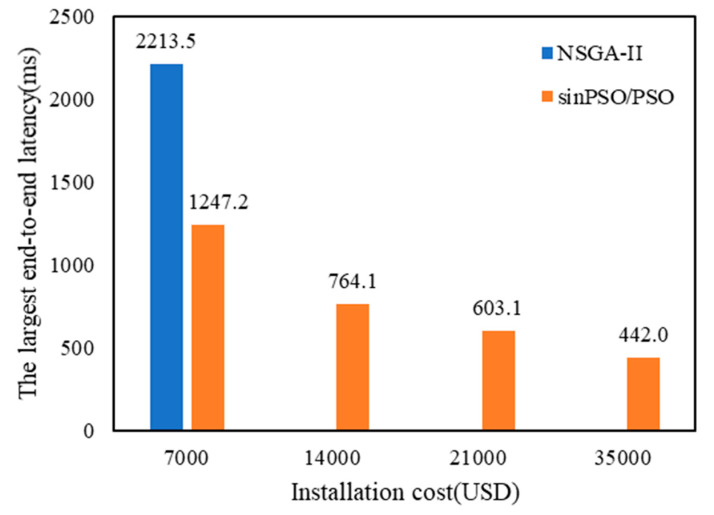
Comparison of deployment schemes based on maximum end-to-end delay and economic optimization.

Analysis of Figure 13 shows that the higher the deployment cost, the more available WCNs, which reduces the number of hops *h* (*p_IR, CMC_*) during patrol data transmission and thus significantly reduces the end-to-end delay. By increasing deployment costs, network delays can be effectively reduced. When solving the layout scheme of Equation (23), due to the small number of data points, SinPSO and PSO obtain the same scheme, and NSGA-II only obtains one deployment scheme.

**Figure 14:**
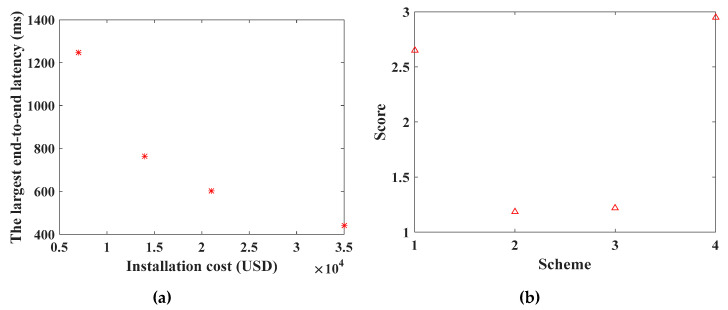
The results of WCN deployment based on SinPSO: (**a**) the Pareto frontier of optimization objective function of WCN installation cost and end-to-end delay; (**b**) the comprehensive attribute score of each deployment scheme of WCN.

When solving the planning scheme of maximum end-to-end delay and deployment cost, the three algorithms are run 100 times. The average time consumed by all algorithms is counted, as shown in Table 7. On the whole, the running time of NSGA-II is significantly longer than that of SinPSO and PSO, and the average running time of SinPSO and PSO is similar. The optimal solution set of Equation (23) Pareto is obtained by SinPSO. The Pareto frontier of the maximum end-to-end delay and deployment cost planning scheme, as shown in Figure 14a, is obtained. Compared with NSGA-II, the maximum end-to-end delay of data transmission between SinPSO and PSO is smaller when the deployment cost is the same, and both SinPSO and PSO are better than NSGA-II in diversity of solution sets.

Among the 4 schemes, the maximum delay is 1247.2 ms, especially when WCNs are densely distributed, and the end-to-end delay of patrol data transmission is 442.0 ms. According to Equation (31), the layout of each scheme is uniform. Using the attribute decision method in Section 3.3, the comprehensive score of each scheme is calculated. As shown in Figure 14b, Scheme 4 has the highest comprehensive attribute score among the four schemes. In Scheme 4, the deployment cost of WCN is USD 35,000, and the maximum end-to-end delay is 442.0 ms. 

The final deployment scenario is shown in Table 8. The tower numbers of WRN deployment location in the final plan are 13th, 53rd, 74th, 118th, 175th, 215th, 238th and 283rd. WCNs deployment locations are 34th, 97th, 149th, 197th and 261st. Thus, the total deployment cost of RHHN is USD 6.3 × 10^4^, the maximum energy consumption of robot is 4.88 × 10^6^ J, and the maximum end-to-end delay is 442.0 ms. From the final deployment result, the deployment locations of communication nodes are evenly distributed, which realizes the real-time hybrid hierarchical network deployment under multiple constraints.

### 4.3. Analysis of Simulation Results 

For RHHN, increasing deployment costs can change energy consumption and end-to-end delays in the data transfer process of the robot. In sensor network applications with different message delivery time requirements, network economy and data timeliness have different requirements. As shown in Figure 8, Figure 9 and Figure 13, the energy consumption of data transmission decreases significantly, and the timeliness of the network increases significantly as the network economy decreases. At the same time, the method can adapt to all kinds of networks with different node energy consumption requirements and data transmission time requirements. It also can improve the controllability of network delay and node energy consumption, and balance the relationship between network economy, efficiency and energy consumption. In view of the application of the robot dynamic monitoring network for the smart gird in this paper, low data transmission delay and a delay controllable network are conducive to improving the level of power grid monitoring. In practical application, a preference matrix can be set reasonably according to actual needs. A network deployment scheme meeting actual needs can be selected to balance economic, real-time and energy consumption of nodes.

## 5. Conclusions

This paper presents a robot hybrid hierarchical network (RHHN) and its optimal deployment method for remote real-time monitoring of transmission lines. Compared with the delay tolerant network proposed in the previous research, the real-time characteristics of RHHN enhance the practicability of the network and improve the monitoring efficiency of the smart grid. The main conclusions are summarized as follows:

(1) According to the characteristics of transmission lines, a hybrid hierarchical network model and architecture for a multi-robot sensing system of smart girds are proposed. The movement law of robot inspection is analyzed, and the calculation method of energy consumption and delay in the process of network data forwarding are given. Then, considering cost, energy consumption, and delay as optimization objectives, a theoretical deployment model of communication nodes is established under multiple constraints.
(2) The relationship between multiple optimization objectives is analyzed, and the multi-objective transformation model is established. The improved PSO is used to solve the deployment scheme. The deployment schemes are sorted by MADM of a vague set with a preference for alternatives, and the optimal deployment scheme is determined according to the actual needs.
(3) In the simulation experiment, the comparison results of several algorithms in a multi-scenario are given. The experimental results show that the energy consumption and delay of the deployment scheme solved by SinPSO are lower under the same deployment cost. At the same time, the diversity of the SinPSO solution set is better, and the time complexity of the algorithm is lower. Taking the scenario with 300 towers as an example, a series of specific RHHN deployment schemes are given by combining the SinPSO algorithm with the MADM.

In this paper, the impact of network node failure on the overall network performance is not considered. As part of future work, we plan to study a cost-efficient fault-tolerant network design.

## Figures and Tables

**Table 1:** The corresponding relationship between parameter *r_ij_* and the vague value.

Interval of Parameter *r_ij_* (%)	Vague Value
[95,100]	[1,1]
[85,95)	[0.9,0.95]
[75,85)	[0.8,0.9]
[65,75)	[0.7,0.85]
[55,65)	[0.6,0.8]
[45,55)	[0.5,0.5]
[35,45)	[0.4,0.6]
[25,35)	[0.3,0.45]
[15,25)	[0.2,0.3]
[0,15)	[0.1,0.15]

**Table 2:** Default parameters for simulations.

Parameter	Default Value
The size of the message received by the robot (B)	30
The size of the message sent by the robot (Kb)	1200
Communication radius of robot, WCN and WRN (m)	8000
Horizontal lobe angle of antenna (°)	60
*E_elec_* (J/bit)	50.0 × 10^−9^
*ε_mp_* (J/(bit·m^4^))	1.3 × 10^−15^
*ε_fs_* (J/(bit·m^4^))	1.0 × 10^−11^
*Ψ* (m/s)	3.0 × 10^8^
*t_ca_* (ms)	41
*C_w_* (USD)	7000
*C_R_* (USD)	3500
*B_ij_* (Mbps)	10
Number of power towers	50/100/200/300/500
Maximum distance between towers (m)	1000
Minimum distance between towers (m)	100

**Table 3:** Parameters of the specific improvement of the improved particle swarm optimization (SinPSO) and PSO algorithm.

Parameter	Value
Population size, *NumEsp*	200
Max Iterations, *MaxIt*	1000
Initial velocity of particles, *v*	0
Particle length, *D*	50/100/200/300/500
Learning factor, *c*_1*max*_	1.2
Learning factor, *c*_1*min*_	0.5
Learning factor, *c*_2*max*_	1.2
Learning factor, *c*_2*min*_	0.5
Maximum inertia weight, *W_max_*	0.45
Minimum inertia weight, *W_min_*	0.1

**Table 4:** Parameters of the non-dominated sorting genetic algorithm II (NSGA-II algorithm).

Parameter	Value
Population size, *N*	200
Length of chromosome, *L*	50/100/200/300/500
Max iterations, *MaxIt*	1000
Recombination probability, *Pc*	0.8
Mutation probability, *Pm*	0.6

**Table 5:** Algorithm running time.

Number of Towers	SinPSO	PSO	NSGA-II
50	12.672 s	12.778 s	25.374 s
100	21.456 s	21.481 s	29.705 s
200	30.169 s	31.195 s	43.048 s
300	36.095 s	36.610 s	49.092 s
500	52.665 s	55.305 s	61.465 s

**Table 6:** The specific layout of Scheme 1 and the distance between communication nodes.

Tower Number of Installation Communication Node	Distance between Communication Nodes (m)
13	8,791.53 (Distance from the first tower)
34	12,405.70
53	11,027.46
74	11,276.60
97	12,981.63
118	11,033.42
149	14,958.80
175	12,767.72
197	12,136.55
215	11,223.56
238	10,698.31
261	12,538.49
283	12,889.18
	9,737.39 (Distance from the last tower)

**Table 7:** Algorithm running time.

	SinPSO	PSO	NSGA-II
Average running time	3.7598 s	3.8231 s	17.4820 s

**Table 8:** Communication network deployment scheme of the inspection robot.

Deployment Plan	Deployment Costs (USD)	Robot Energy Consumption (J)	Maximum End to End Delay (ms)
13th, 34th *, 53rd, 74th, 97th *, 118th, 149th * 175th, 197th *, 215th, 238th, 261st *, 283rd	6.3 × 10^4^	4.88 × 10^6^	442.0

Note: x* is the installation location of WCN, x is the installation location of WRN, and x stands for tower number.
